# Janina Hurynowicz (1894–1967)

**DOI:** 10.1007/s00415-020-10259-x

**Published:** 2020-10-14

**Authors:** Anita Magowska, Piotr Skalski

**Affiliations:** Poznan University of Medical Sciences, Poznan, Poland


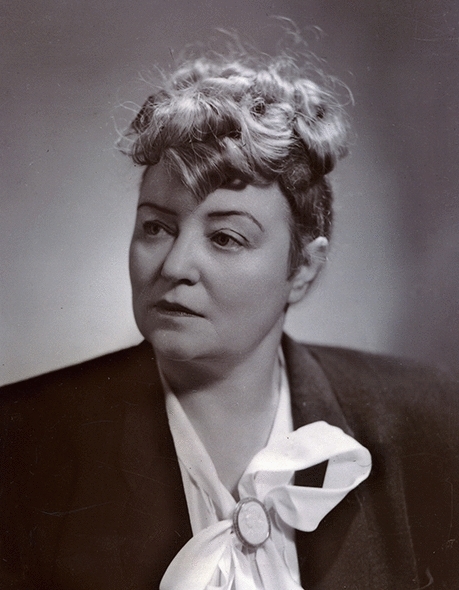


One of the first women to take up the study of neurology was Janina Hurynowicz, a Polish physician. Hurynowicz contributed to the development of neurology by research based on rheobase (the lowest current intensity required to cause a response) and chronaxie (the shortest time, in milliseconds, required to produce the same reaction with double the rheobasic strength) measurements. The parameters were introduced in 1909 by Louis Lapicque (1866–1952) to quantify nerve excitability [1]. Interest in chronaximetry peaked in the 1970s, however, the method is still in use today in experimental and clinical studies [2].

Hurynowicz’s life story is a tale of the beginnings of neuromuscular electrodiagnostics, but is also an account of a woman’s hampered career in neurology. Born in 1894 near Vilnius, in the territory of the Polish–Lithuanian Commonwealth annexed by Russia, Hurynowicz faced a higher education system closed to women. To receive a school-leaving certificate, she had to pass the matriculation examination as an extramural student at a secondary school for boys.

In 1911–1918, she studied medicine at the Female Medical Institute in St. Petersburg in Russia; however, due to the turmoil of the Russian Revolution of 1917, Hurynowicz graduated in the new Soviet Union. Now with a doctor’s diploma, she was assigned to work successively as a surgeon, epidemiologist, and neurologist in the Russian Civil War hospitals. Nevertheless, when a Polish division was formed in Siberia to fight the Bolsheviks, she joined it and, despite her young age and being the only woman among thousands of soldiers, became the head of a field hospital. In January 1920, along with the division, Hurynowicz was taken captive by the Bolsheviks and directed to work as a neurologist at a hospital for prisoners of war in Verkhne-Udinsk, the capital of the Far East Republic, a puppet and ephemeral state created by the Soviets to avoid an open conflict with Japan. With the Red Cross, she was evacuated to Japan and, via India, France and Germany, arrived in Vilnius in an already independent Poland.

She was still a military doctor, so she was appointed the neurological ward's chief physician at the Vilnius military hospital. Demobilized in September 1921, she took up a post in a neurological dispensary for children and in another, organized by the local insurance company for patients with neurological pathologies. In the following year, she resigned because she was starting her scientific career as an assistant at the Neurological Clinic of the Faculty of Medicine at the University of Vilnius.

Five years later, Hurynowicz had obtained a doctor’s degree. Her dissertation on the influence of insulin on the vegetative nervous system (today known as the autonomic nervous system, ANS) was rated so highly that the university authorities decided to send her to Paris to learn about chronaxie measurement and other new trends in neuroscience. She studied in the Salpêtrière and Pitié hospitals under such eminent neurologists as Joseph Babinski (1857–1932), Georges Guillain (1876–1961) and Jean-Athanase Sicard (1872–1929), as well as the neurosurgeon Clovis Vincent (1879–1947) and the psychiatrist Maxime Laignel-Lavastine (1875–1953). At the same time, she explored the theory and technique of measuring nerve excitability in the laboratory of Louis Lapicque at the Sorbonne and was introduced to its clinical application by Alfred Georges Bourguignon (1876–1963). The method of chronaximetry was of great importance to the neurology of the time because it provided the first parameters for quantifying nerve excitability [3].

Soon after returning from Paris, Hurynowicz received a *venia legendi* in neurology based on her habilitation thesis on the chronaxie of iterative nerves, which, according to Lapicque, had different functions; for example, the vagus nerve interfaces with the parasympathetic control of the heart, lungs and digestive tract. In 1933, she unexpectedly lost her position because the neurological clinic was reorganized into a clinic for nervous and mental diseases that was entrusted to Maksymilian Rose (1883–1937) [4]. Hurynowicz had to move to the Department of Physiology to run its Neurophysiology Laboratory. Four years later, she had to break her research again because the University Senate requested that she manage the clinic of nervous and mental diseases after Rose's death until the right candidate for the position of head was found. It was a sign of the times; despite Hurynowicz being a talented and experienced neurologist, a woman would never have been acceptable in a clinical job. In 1938, Hurynowicz applied for a similar position at the University of Poznan, also unsuccessfully.

On 17 September 1939, the Russians occupied Vilnius but in December handed the city over to the Lithuanians, who immediately closed the Polish University. Hurynowicz lost her position and had to take a job in the municipal neurological hospital as its Deputy Director. She became actively involved in the Polish Underground State and risked her life to treat wounded partisans.

After the war, Vilnius remained within the borders of the Lithuanian People’s Republic, so Hurynowicz decided to repatriate to Poland and settled in Torun, where the Nicolaus Copernicus University had just been established. As an associate professor, she first organized and then became the Head of the Department of Neurophysiology and Comparative Physiology at the University and of the local mental health clinic in the same building. Moreover, she was the Vice-Dean of the Faculty of Mathematics and Natural Sciences at the University of Copernicus, a lay judge, a trainer in the method of chronaximetry for young researchers, and a lecturer on neurological pathologies for doctors, nurses and teachers. In 1949, she became a full professor. Simultaneously, the state authorities ordered her to set up and temporarily run a physiology department in the Faculty of Medicine at the newly established Gdansk School of Medicine, which required her to commute to a distant town.

Living during the Cold War, she was not able to develop international cooperation, but participated in a few congresses abroad. Fascinated by French culture, Hurynowicz belonged to the Societé Philomatique and the Association de la Langue Française. Apart from other societies, she was also a member of the German Society of Neurologists and the Torun Medical Society, which she founded. She did not start a family. Retired at the age of 70, she died on 2 October 1967, in Torun [5].

Her most important contributions to neurology were based on studies made during her stay in France. Together with A. B. Chauchard, Hurynowicz improved Lapicque’s device to enable the separate measurements of the chronaxie of vasomotor nerves and the vessel innervated by them [6]. In turn, using the device with modifications by Bourguignon, which enabled measurements through the skin, she studied the reflexes related to the activity of individual parts of the labyrinth, that is, the utriculus, sacculus, and semicircular canals. The results were useful for aviation medicine [7]. She found new experimental and clinical applications for the measurements of chronaxie. For example, she demonstrated that in cases of physiological weariness, the nervous system is a coordinated mechanism with a uniformly reduced excitability of its components. In fatigue, this mechanism is distracted, mostly due to the cerebellum and its reaction with the reticular formation [8]. Hurynowicz proved that chronaximetry might be a crucial method for studying the peripheral vasomotor system and monitoring therapeutic results in orthopaedic surgery [9]. Her research on the processes within the nervous system under the influence of hypoxia, hypoglycaemia and anaphylactic shock were also of clinical importance [10].
